# Ursolic Acid Protects Sodium Dodecyl Sulfate-Induced *Drosophila* Ulcerative Colitis Model by Inhibiting the JNK Signaling

**DOI:** 10.3390/antiox11020426

**Published:** 2022-02-21

**Authors:** Tian Wei, Lei Wu, Xiaowen Ji, Yan Gao, Guiran Xiao

**Affiliations:** 1 Key Laboratory of Metabolism and Regulation for Major Diseases of Anhui Higher Education Institutes, School of Food and Biological Engineering, Hefei University of Technology, Hefei 230009, China; weitfield@126.com (T.W.); 18605666360@163.com (L.W.); jxwjyl@163.com (X.J.); 13095531206@163.com (Y.G.); 2 Department of Toxicology, School of Public Health, Anhui Medical University, Hefei 230032, China

**Keywords:** ursolic acid, ulcerative colitis, JNK, JAK/STAT, *Drosophila*

## Abstract

Ursolic acid (UA) is a bioactive molecule widely distributed in various fruits and vegetables, which was reported to play a therapeutic role in ulcerative colitis (UC) induced by toxic chemicals. However, the underlying mechanism has not been well clarified in vivo. Here, using a *Drosophila* UC model induced by sodium dodecyl sulfate (SDS), we investigated the defensive effect of UA on intestinal damage. The results showed that UA could significantly protect *Drosophila* from the damage caused by SDS exposure. Further, UA alleviated the accumulation of reactive oxygen species (ROS) and malondialdehyde (MDA) induced by SDS and upregulated the activities of total superoxide dismutase (T-SOD) and catalase (CAT). Moreover, the proliferation and differentiation of intestine stem cells (ISCs) as well as the excessive activation of the c-Jun N-terminal kinase (JNK)-dependent JAK/STAT signaling pathway induced by SDS were restored by UA. In conclusion, UA prevents intestine injury from toxic compounds by reducing the JNK/JAK/STAT signaling pathway. UA may provide a theoretical basis for functional food or natural medicine development.

## 1. Introduction

Ulcerative colitis (UC) is a chronic and recurrent inflammatory bowel disease that is listed as one of the modern refractory diseases by the World Health Organization (WHO) [1]. UC is characterized with non-specific chronic inflammation of the intestine, which causes various complications, such as increased risk of colorectal cancer [2]. However, some limitations exist in the present therapies, such as unsatisfactory long-term efficacy, drug resistance, and severe systemic side effects [3,4]. Therefore, novel effective and sustained treatments are urgently needed. The single-layered simple epithelium of the gastro-intestinal tract controls nutrient uptake, coordinates our metabolism and shields us from luminal components including chemical poison or pathogens [5,6]. Therefore, the maintenance of intestinal barrier function, including its metabolic and immune functions, is essential for organismal health. Epithelial homeostasis is dependent on a balance of intestinal stem cell (ISC) self-renewal, progenitor differentiation, cell shedding and apoptosis [7]. In recent years, more and more studies are focused on how different nutritional states influence ISC function and epithelial homeostasis.

Ursolic acid (UA) is an anti-inflammatory natural triterpenoid found in large quantities in different fruits and vegetables, including apple, basil, cranberry, and peppermint [8,9]. Several studies have shown that UA regulates numerous biological processes including antioxidant [10], anti-cancer [11], anti-inflammation [12], anti-bacterial [13] and immunomodulatory processes [7]. UA was recently reported to play a crucial role in intestinal protection [14,15]. The chemical compounds sodium dodecyl sulfate (SDS) and dextran sulfate sodium (DSS) have previously been shown to induce epithelial cell damage [16]. Therefore, they are widely used to induce UC in many model organisms [17,18,19]. UA was reported to protect a DSS-induced mouse UC model by reducing the upregulation of NF-κB in the colon tissues [20]. However, the underlying mechanism of UA restoring UC needs further investigation. New insights have demonstrated that multiple metabolic signaling pathways are involved in the regulation of SDS- or DSS-induced intestinal disruption, such as the c-Jun N-terminal kinase (JNK) [21,22], JAK/STAT, epidermal growth factor receptor (EGFR), and Wnt and Notch pathways [23,24]. UA was previously reported to repress tumor growth through the JNK and JAK/STAT pathways in human cancer cells [17,25], so we wondered whether these pathways are involved in the protection of UA against the intestine damage of UC.

*Drosophila melanogaster* (hereinafter *Drosophila*) represents one of the most important model organisms and has made fundamental contributions to different areas of biology [26]. The digestive system of *Drosophila* contains the foregut, proventriculus, midgut and hindgut, and the intestinal function of *Drosophila* is similar to that of mammals [27,28]. The *Drosophila* midgut harbors multipotent adult stem cells that are essential to renew the gut in homeostatic conditions and upon stress-induced regeneration, and the *Drosophila* midgut has emerged in recent years as a model system to decipher regulatory mechanisms of stem cell biology [29,30]. Comparative genomic studies estimate that up to 75% of the human genes implicated in diseases are conserved in *Drosophila* [31]. In addition, the signaling pathways involved in development of diseases, such as the JNK, JAK/STAT, Wnt and Notch pathways, are highly conserved from *Drosophila* to human beings [32,33]. Therefore, *Drosophila* has become a powerful model to study intestinal physiology and pathology [32]. For example, *Drosophila* has been used to screen plant extracts with potential applications in many chemical-induced intestinal disorders [34,35].

In this study, *Drosophila* was employed to investigate the effect and regulatory mechanism of UA on the intestine disruption induced by SDS. The results indicated that the decreased survival rate and mobility of adult flies caused by SDS could be restored by UA. In addition, the increased reactive oxygen species (ROS) and malondialdehyde (MDA) levels caused by SDS could be reduced by UA. Further studies indicated that the activated JNK and JAK/STAT pathways induced by ROS in UC models could be rescued by UA. These results elucidate the protective effect of UA on UC caused by environmental stresses. This study is of great significance for UA application and may improve the medicinal and nutritional value of UA in the future.

## 2. Materials and Methods

### 2.1. Materials and Reagents

The UA (purity ≥ 98%) was purchased from Aladdin Co., Ltd. (#U118635, Shanghai, China). The SDS (#L4509) and Brilliant Blue FCF (#80717) were purchased from Shanghai Macklin Biochemical Co., Ltd. (Shanghai, China). The phosphorylated JNK (pJNK, Cat#07-175) antibody and Triton X-100 (#T8787) were purchased from Sigma-Aldrich Trading Co., Ltd. (Shanghai, China). The Cy3-conjugated goat anti-rat IgG (#A0521), 2-(4-Amidinophenyl)-6-indolecarbamidine dihydrochloride (DAPI, #C1005), phosphate buffered saline (PBS, #ST476), total superoxide dismutase (T-SOD) assay kit (#S0101), catalase (CAT) assay kit (#S0051) and lipid peroxidation (MDA) assay kit (#S0131) were purchased from Beyotime Biotechnology (Shanghai, China). The 2′,7′-dichlorodihydrofluorescein diacetate (H_2_DCFDA) was purchased from Thermo Fisher Scientific Co., Ltd. (Shanghai, China). All other reagents used were of analytical grade and commercially available.

### 2.2. Fly Husbandry

The following flies were used: w1118 (V#60000) was obtained from the Vienna *Drosophila* RNAi Center (Vienna, Austria). *esg*-Gal4; UAS-GFP; *tub*-Gal80^ts^ (*esg*-GAL4^ts^) and *Myo1A*-Gal4; UAS-GFP; *tub*-Gal80^ts^ (*Myo1A*-GAL4^ts^) flies were kindly gifted from Dr. Lihua Jin (Northeast Forestry University, Harbin, China) [36]. 10×STAT-GFP was generously provided by Dr. Jose C. Pastor-Pareja (Tsinghua University, Beijing, China) [37]. The *esg*-GAL4^ts^ and *Myo1A*-GAL4^ts^ flies were collected within 48 h after eclosing and aged for 5 days before shifting to 29 °C (restrictive temperature for Gal80^ts^) to induce GFP expression. Animals of the other genotypes were cultured under 25 °C and 60% humidity with a 12 h light:12 h dark cycle, unless otherwise noted. All flies were cultured on corn-yeast standard food as the previous report [38]. The concentration of UA used in vivo was slightly adjusted according to Staats’s research [18]. 

### 2.3. Survival Assays

For survival experiments, groups of ~3 days newly emerged flies (40 females and 40 males) reared on normal food (NF) or UA-containing food (100 μM) were starved for 2 h before being transferred into vials containing three layers of filter paper soaked with 200 μL of 5% sucrose (control group) or 5% sucrose solution containing 0.5% SDS (NF-SDS, UC model group). To summarize, the control group flies were emergenced on normal food for 7 days and then fed with 5% sucrose (NF-SUC). The UC model group flies were emergenced on normal food for 7 days and then fed with 5% sucrose supplemented with 0.5% SDS (NF-SDS). The experimental group flies were emergenced on 100 μM UA food for 7 days and then fed with 5% sucrose supplemented with 0.5% SDS (UA-SDS). The filter papers and solution were changed once every 24 h. The surviving flies were counted and recorded every day. The experiment was repeated three times.

### 2.4. Climbing Ability Assay

Climbing ability assay was performed as described previously [39]. Ten newly emerged (~3 day old) flies were maintained on UA-supplemented (100 μM) or control diet for 7 days. Then, the flies exposed to sucrose or SDS (0.5%, *w*/*v*) for 24 h (as stated in Section 2.3), were collected and placed in plastic cylinder (1.5 cm diameter × 16 cm length). The flies were gently hit to ensure all flies down to the bottom of the plastic cylinder. The climbing ability was evaluated by the proportion of flies that climbed upwards more than 10 cm within 10 s.

Climbing ability assay was performed as described previously [39]. Ten newly emerged (~3 day old) flies were maintained on UA-supplemented (100 μM) or control diet for 7 days. Then, the flies exposed to sucrose or SDS (0.5%, *w*/*v*) for 24 h (as stated in Section 2.3), were collected and placed in plastic cylinder (1.5 cm diameter × 16 cm length). The flies were gently hit to ensure all flies down to the bottom of the plastic cylinder. The climbing ability was evaluated by the proportion of flies that climbed upwards more than 10 cm within 10 s.

### 2.5. Intestinal Morphology Analysis

Twelve newly eclosed (~3 day old) female flies were maintained on UA-supplemented (100 μM) or control diet for 7 days and then exposed to sucrose or SDS (0.5%, *w*/*v*) for 48 h (as stated in Section 2.3), were dissected in the 1× PBS and immediately observed under a Nikon ECLIPES Ti2-U microscope (Nikon, Tokyo, Japan)

### 2.6. Smurf Assay

The Smurf assay was performed as previously described with minor modifications [18]. Briefly, newly eclosed female flies were maintained on UA-supplemented (100 μM) or control diet for 7 days. Then, the flies were treated with SDS (0.5% SDS in 5% sucrose, *w*/*v*) or 5% sucrose as negative control (*w*/*v*). For the assay, the flies were starved for 2 h every other day and then treated for 6 h with the SDS/sucrose solution soaked on a Whatman filter paper before they were relocated to Brilliant Blue FCF-dyed food. After feeding for 7 days, the flies were frozen to death, photographed and then homogenized with 1× PBS and centrifuged. The intestine integrity was determined by the absorbance of the supernatant at 625 nm as the previous reports [18,40].

### 2.7. Reactive Oxygen Species Assay

Adult females were exposed to SDS (0.5%, *w*/*v*) and incubated at 25 °C for 48 h. A total of 10~15 intestines were dissected in cold 1× PBS and incubated in H_2_DCFDA (5 µM in PBS) for 10 min, then washed 3 times in cold 1× PBS for 5 min, and immediately observed under a Nikon ECLIPES Ti2-U microscope (Nikon, Tokyo, Japan). The data presented are from three independent experiments.

### 2.8. Immunohistochemistry

For pJNK staining in intestines, the intestines of female flies were dissected, fixed, stained, and mounted following standard procedures [41,42]. The following antibodies were used: rabbit anti-pJNK (1:200) and Cy3-conjugated goat anti-rat IgG (1:500). For STAT testing in intestines, the intestines of 10× STAT-GFP flies were dissected and immediately fixed in 4% paraformaldehyde and washed three times with 0.3% PBST (PBS containing 0.3% Triton X-100). For nucleic acid staining, samples were incubated in 50 ng/mL DAPI for 8 min. Slices were mounted with 50% glycerol/PBS. Confocal images were taken with a Zeiss LSM710 Meta confocal microscope. ImageJ was employed to quantify the pJNK and STAT-GFP level.

### 2.9. Fluorescence Microscopy

Intestines of *esg*-GAL4^ts^ and *Myo1A*-GAL4^ts^ flies were dissected and immediately fixed in 4% paraformaldehyde, stained, and mounted. Slices were mounted with 50% glycerol/PBS. Quantification of GFP intensity in the intestine was constructed by measuring GFP^+^ cell fluorescence intensity within posterior midgut [36]. Fluorescence of GFP^+^ cells was measured using ImageJ.

### 2.10. Statistical Analysis

Data were analyzed by using Student’s t-test to compare between groups and one-way analysis of variance (ANOVA) for multiple groups. The statistical results were presented as mean ± SEM. Asterisks indicate the critical levels of significance (* *p* < 0.05, ** *p* < 0.01, and *** *p* < 0.001).

## 3. Results

### 3.1. The Intestinal Disruption Induced by SDS in Drosophila Could Be Rescued by UA

**Figure 1:**
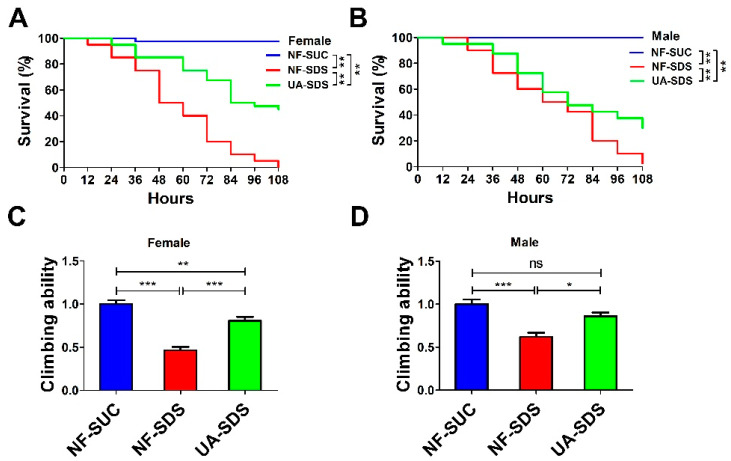
The reduced survival rate and climbing ability caused by sodium dodecyl sulfate (SDS) could be improved by ulcerative colitis (UA) in *Drosophila*. (**A**,**B**) The ulcerative colitis (UC) model group (NF-SDS) showed shorter life span than control group (NF-SUC). The 100 μM UA supplementation (UA-SDS) can significantly elevate the decreased survival rates of both female (**A**) and male (**B**) flies exposed to 0.5% SDS (NF-SDS). (**C**,**D**) The UC model group (NF-SDS) showed decreased climbing ability compared to the control group (NF-SUC). UA supplementation (UA-SDS) elevated the reduced climbing ability of female (**C**) and male (**D**) flies treated with 0.5% SDS. * *p* < 0.05, ** *p* < 0.01, *** *p* < 0.001, ns: not significant.

To investigate the effect of UA on chemical-induced UC, we produced a *Drosophila* UC model induced by the inflammatory reagent SDS (NF-SDS). The UC model group flies (NF-SDS) showed shorter life spans and decreased mobility compared with the control group (NF-SUC) (Figure 1A–D). As shown in Figure 1A,B, flies which were pre-fed with 100 μM UA (UA-SDS) showed a significant resistance to SDS. Following treatment with SDS for 108 h, the survival rates of female and male flies were dramatically increased from 0% to 45% (*p* < 0.001) and from 2.5% to 30% (*p* < 0.001) in the UA-SDS group, respectively, compared with the NF-SDS group (Figure 1A,B). In addition, SDS exposure induced significant reduction of climbing ability (female down 54%; male down 38%); the decreased climbing abilities were weaker in UA-SDS flies (female, showing a 35% increase; male, showing a 24% increase) (Figure 1C,D). These results indicated that UA could increase the survival rate and climbing activity of flies treated with toxic chemical SDS. Taken together, UA showed protection against chemical-induced UC in *Drosophila*.

### 3.2. Protective Effect of UA against Morphological Changes in the Drosophila Intestine following Treatment with SDS

**Figure 2:**
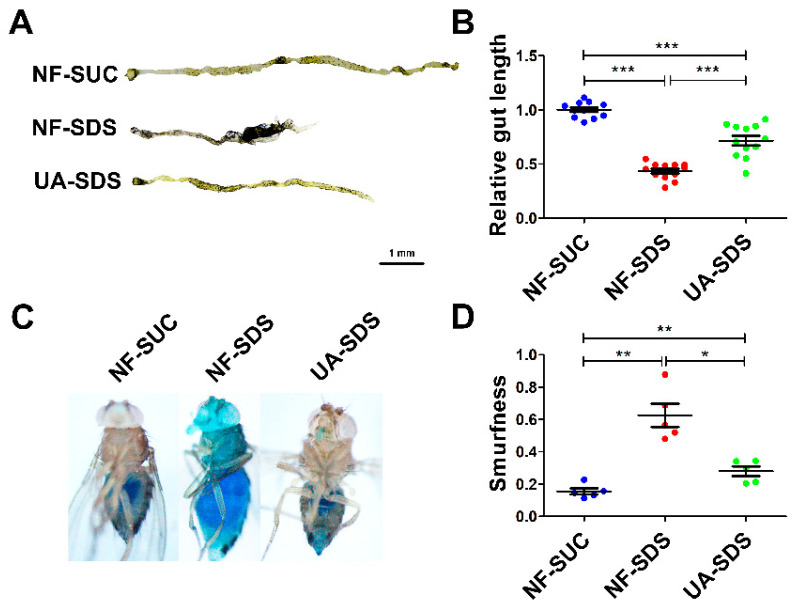
UA alleviated SDS-induced impairment of intestinal morphology and barrier integrity. (**A**) After exposure to 0.5% SDS for 48 h, the intestines of these flies (NF-SDS) appeared shorter than control (NF-SUC), UA significantly increased the intestine length. (**B**) Quantitative measurement of (**A**) (n ≥ 10). (**C**) A small amount of blue dye occurred in the gut of control flies (NF-SUC) while the blue dye escaped throughout the whole body of UC model flies exposed to 0.5% SDS for 48 h (NF-SDS), which can be prevented by pre-feeding of UA (UA-SDS). (**D**) The intestinal barrier permeability was quantified by measuring the Smurfness (n = 5). * *p* < 0.05, ** *p* < 0.01, *** *p* < 0.001.

The male flies and female flies upon SDS exposure exhibited largely similar phenotypes, except that the phenotypes of the female flies were more severe than those of the male flies. In addition, the protection of UA on female flies against SDS was better than the males. Therefore, only the results of female flies are shown hereafter. As shown in Figure 2A,B, the intestines of UC model flies (NF-SDS) appeared shorter by 57% than the control (NF-SUC). These results were consistent with previous studies [34,35]. Compared with the UC model group, the intestine length of flies in the UA-SDS group was extended by 64% (*p* < 0.001). In addition, the intestinal epithelium is susceptible to damage caused by the chemical compound SDS [35]. SDS-induced colitis showed disrupted intestinal barrier integrity [34,43]. The intestinal barrier function could be evaluated by using a blue dye, Brilliant Blue FCF. Loss of intestinal barrier function showed the Smurf phenotype with blue dye throughout the whole body of flies, which could be quantified by Smurfness [44,45]. To investigate the protective effect of UA on UC, the Smurf assay was used to evaluate intestine integrity of flies. After exposure to SDS, the flies showed enhanced blue distribution when compared to the control, showing a 304% increase in Smurfness (Figure 2C,D). This suggests that exposure to SDS increased intestinal permeability, indicating damage to the epithelial barrier. UA supplementation alleviated the epithelial damage, even to the normal level (Figure 2C,D). Taken together, our findings suggested that UA protects against disruption of the intestinal barrier caused by SDS in *Drosophila*.

### 3.3. UA Protected Intestinal Stem Cells from SDS-Induced Proliferation and Differentiation

**Figure 3:**
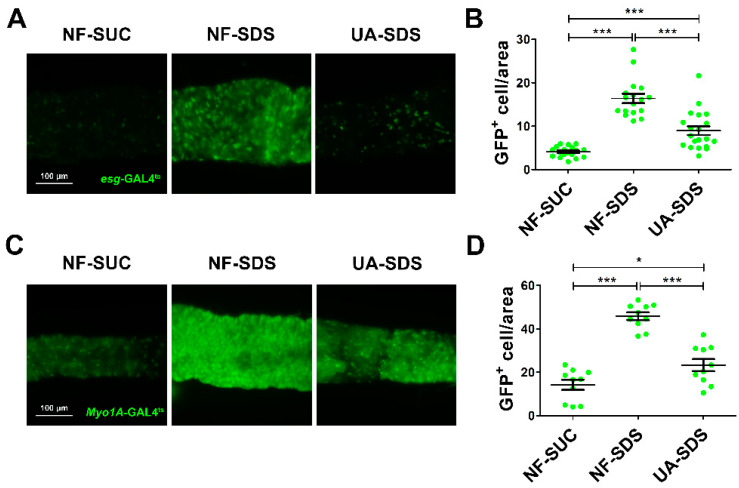
UA protected intestine from SDS-induced intestinal stem cell (ISC) proliferation and differentiation. (**A**) UA could significantly reduce proliferation and differentiation of ISCs/ enteroblasts (EBs). (**B**) Fluorescence intensity of the GFP^+^ ISCs/EBs in panels (n ≥ 15). (**C**) UA could strongly decrease SDS-induced EC proliferation. (**D**) Fluorescence intensity of the GFP^+^ EC cells in panels (n ≥ 10). * *p* < 0.05, *** *p* < 0.001.

Intestinal damage caused by chemicals results in the activation of ISC proliferation to regenerate the damaged intestinal epithelium [46]. To evaluate the protective effect of UA on the intestinal homeostasis, SDS was employed to stimulate the proliferation and differentiation of ISCs [16]. The numbers of ISCs/enteroblasts (EBs) were detected by GFP expression driven by *esg*-Gal4^ts^ (as revealed by fluorescence intensity of GFP^+^ cells) [47]. After 48 h of exposure to SDS, the GFP^+^ cells were clustered and showed aberrant morphology (Figure 3A). A statistically dramatic increase in ISCs/EBs was observed in the NF-SDS group, showing a 4-fold increase in fluorescence intensity when compared to the control group (Figure 3B). UA supplementation clearly restored the aberrated morphology of ISCs/EBs and showed a strong reduction in the number of ISC and EB cells (down 44%) (Figure 3A,B).

*Myo1A* is specifically expressed in enterocytes (ECs) [48]. Subsequently, we used the GFP level of *Myo1A*-GAL4^ts^ to analyze the numbers of ECs. As shown in Figure 3C,D, we found that SDS could promote EBs to further differentiate into ECs in the NF-SDS group (a 2.2-fold increase in fluorescence intensity of *Myo1A* GFP^+^ cells, compared with the control group). As expected, the increased ECs in the NF-SDS group could be significantly attenuated by UA; however, the number of ECs in the UA-SDS group remained larger than the control group (*p* < 0.05). These results indicated that the ISCs proliferation and differentiation induced by SDS could be rescued by UA.

### 3.4. UA Protects Intestine against SDS-Induced Oxidative Damage

**Figure 4:**
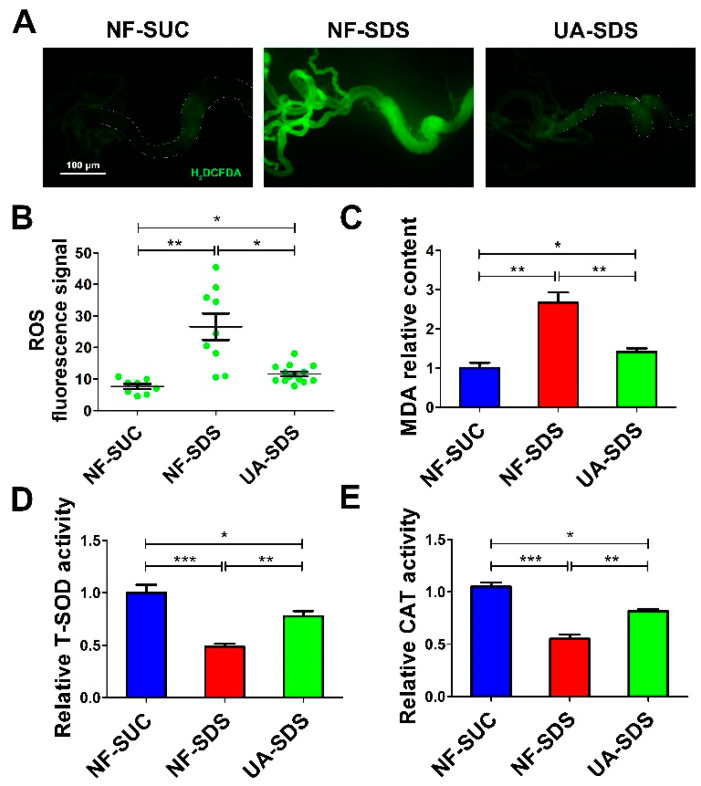
UA protected intestine from SDS-induced oxidative damage. (**A**) UA protected epithelial cells against SDS-induced oxidative stress. H_2_DCFDA staining of the anterior midgut of a female fly after ingestion of 0.5% for 48 h. (**B**) Fluorescence intensity of ROS levels in panels (n ≥ 10). (**C**–**E**) UA supplementation alleviated malondialdehyde (MDA) levels and increased the activities of total superoxide dismutase (T-SOD) and catalase (CAT), after exposure to SDS for 48 h. * *p* < 0.05, ** *p* < 0.01, *** *p* < 0.001.

Environmental stresses cause excessive ROS, which induces cellular oxidative damage [49,50], ISC activation and tissue renewal [51]. Recent studies revealed that UA could significantly alleviate the ROS production and cell death induced by a mycotoxin ochratoxin A [52]. Then, we tested whether UA could attenuate the excessive ROS levels in the damaged intestine. H_2_DCFDA, a cell-permeable probe, was used to detect the intracellular ROS levels. Adult flies were exposed to SDS for 48 h and a robust fluorescence signal was observed in NF-SDS compared with control. The enhanced fluorescence signal in the posterior midgut of the NF-SDS flies was markedly reduced in the UA-SDS group flies (Figure 4A). Statistical analysis indicated that UA showed a significant attenuation in the fluorescence signal (down 57%, *p* < 0.05) compared with the UC model group (Figure 4B). Additionally, SDS exposure caused a significant increase in MDA, a marker of lipid peroxidative damage [53], showing a 167% increase compared with control. Treatment with UA significantly reversed the SDS-induced MDA accumulation (Figure 4C). These findings suggested that UA protected the adult flies against SDS-induced oxidative damage.

Many antioxidant enzymes, such as superoxide dismutase (SOD) and catalase (CAT), help organisms resist oxidative damage [54]. To further investigate how UA protects against SDS-induced ROS, the activities of these antioxidant enzymes were examined. As shown in Figure 4D,E, the activity of either total SOD (T-SOD) or CAT was strongly downregulated upon SDS exposure (decreased by 52% and 45%, respectively, compared to the control), whereas UA supplementation restored the activities of these enzymes (30% and 27% increase in T-SOD and CAT activity respectively, compared to NF-SDS group). Collectively, the results suggested that UA may maintain the host redox homeostasis following SDS exposure, therefore protecting against chemical-induced ISC proliferation and differentiation.

### 3.5. UA Protects against SDS-Induced Intestine Damage by Regulating JNK Signaling Pathway

**Figure 5:**
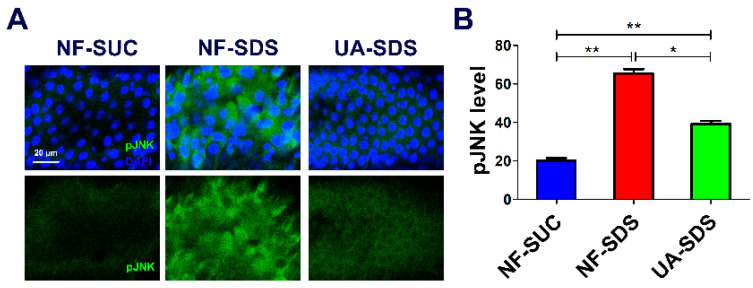
UA alleviated SDS-induced intestine damage by inhibiting c-Jun N-terminal kinase (JNK) signaling. (**A**) Immunohistochemistry indicated that the induced level of phosphorylated JNK (pJNK) as well as the aberrant nucleus morphology caused by SDS were attenuated by UA. (**B**) Quantification of pJNK levels in the intestines (n ≥ 6). * *p* < 0.05, ** *p* < 0.01.

Many studies demonstrated that ROS can activate the JNK signaling pathway, and the ROS/JNK signaling plays a vital role in environmental stress responses [55]. Moreover, the ROS-induced JNK signaling can boost ISC proliferation [56]. To further assess whether the JNK signaling participates in the protection of UA on the intestine, the pJNK levels were detected as an indicator of JNK signaling [57]. After exposure to SDS for 48 h, the posterior intestine of UC model flies (NF-SDS) suffered severe injury, and the expression of pJNK was dramatically increased (a 2.27-fold increase, compared with control) (Figure 5A,B). In addition, we found that SDS treatment led to aberrant nucleus morphology, clustered nuclei and enlarged size (increase of 55%) (Figure S1), which mimics the results in DSS treatment [36]. Compared with the NF-SDS group, UA supplementation showed a significant attenuation in pJNK level (down 40%) and rescued the enlarged nuclear size (Figure 5A,B and Figure S1). Collectively, the results demonstrated that UA may protect the *Drosophila* intestine from SDS-induced damage through the JNK signaling pathway.

### 3.6. JAK/STAT Pathway Is Involved in the Protection of UA on Drosophila Intestine Homeostasis

**Figure 6:**
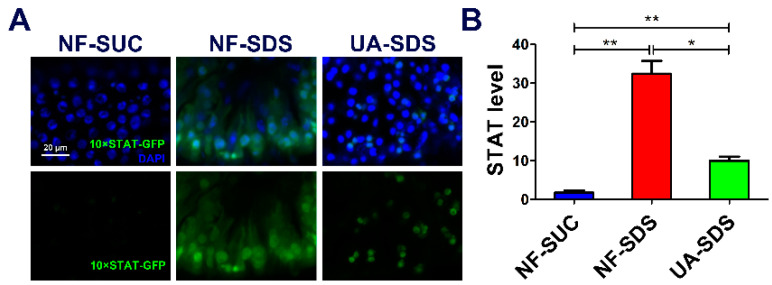
UA protected against SDS-induced injury via regulating JAK/STAT signaling pathway. (**A**) The JAK/STAT signaling was activated by SDS, and UA significantly attenuated the SDS-induced JAK/STAT activation. (**B**) Quantification of 10×STAT-GFP levels in the intestines (n ≥ 6). * *p* < 0.05, ** *p* < 0.01.

JNK acts synergistically with JAK/STAT in response to tissue damage in fly tissues [58]. Intestine damage caused by feeding flies with chemicals stimulates ISC proliferation through the JNK-regulated JAK/STAT pathway [59]. Given that chemical SDS is long known to cause JAK/STAT activation [44], we examined whether the JAK/STAT signaling participates in UA’s protective mechanism. As shown in Figure 6A,B, the STAT activity was obviously activated in the NF-SDS group (feeding SDS for 48 h) as monitored by expression of 10×STAT-GFP (a 17.7-fold increase), compared with the control group. Pre-supplemented UA could alleviate the upregulated expression of 10×STAT-GFP [37] (down 69%) (Figure 6A,B). These data suggested that UA may regulate the JNK/JAK/STAT responsive module to maintain *Drosophila* intestine homeostasis from toxic chemical-induced disruption.

## 4. Discussion

The intestinal epithelium is a prominent defense barrier against environmental stresses [60], and the disruption of intestinal integrity is one of the primary causes of several intestinal disorders. Herein, we investigated the function of UA in protecting against SDS-induced intestine damage. The results demonstrated that pre-supplemented UA significantly increased the survival rates and locomotor activity of flies cultured on SDS. Moreover, UA could restore the ISC proliferation and differentiation induced by SDS. Further study indicated that the protection of UA on *Drosophila* intestine homeostasis is mediated by the reduction of the JNK/JAK/STAT signaling pathway. These results provide in vivo evidence for the protective effect of UA on SDS-induced intestinal dysfunction. Therefore, our findings may provide a theoretical basis for functional food or natural medicine development of UA.

ROS plays an important role in maintaining intestinal homeostasis and improving stress tolerance in organisms [49]. Excessive ROS have been implicated in toxic chemical-induced intestinal disorders [61]. Enterocytes sense ROS and then regulate the proliferation and differentiation of ISCs and therefore maintain intestinal health and homeostasis [16,62]. Here we found that SDS significantly increased the ROS-induced ISC cell proliferation and differentiation (Figure 4A,B), which was consistent with Zhang’s research that DSS induced the ISC cell proliferation and differentiation [36]. It has been reported that UA could reduce ROS generation in mesangial cells and alleviated mesangial cell injury [19]. In addition, UA elevated the activities of T-SOD and CAT responsible for scavenging ROS (Figure 4D,E). Therefore, we concluded that UA may increase the activities of several ROS-scavenging antioxidant enzymes and regulate the SDS-induced ISC proliferation and differentiation.

Several signaling pathways have been implicated in maintaining intestinal homeostasis, such as JNK, JAK/STAT, Notch and Hippo pathways [63,64,65]. Furthermore, a coordinated activation of conserved signaling modules, such as JNK and JAK/STAT, plays crucial roles in tissue restoration in response to environmental stress and injury [66]. For example, the crosstalk between JNK and Hippo signaling regulates ISC proliferation response to stress [63,67]. Previous studies have shown that the activation of JNK signaling caused by environmental stresses in ECs can induce cytokine upd3 expression, which triggers the JAK/STAT pathway required for ISC proliferation in intestinal regeneration [44,68]. In this study, we found that UA could inhibit the JNK signaling by attenuating the phosphorylation of JNK and then reducing the SDS-induced JAK/STAT activation (Figure 6A,B). Therefore, we proposed that UA may protect UC by restoring the ISC proliferation and ROS production by inhibiting the JNK/JAK/STAT pathway and enhancing the resistance of *Drosophila* to environmental challenges. It is unknown whether there are other pathways that participate in this process. Additional protective molecular pathways stimulated as response to UA may be further explored by using high-throughput transcriptome-wide RNA sequencing in the future. Even though *Drosophila* has many organs that function like mammalian organs [32], there are some morphological differences. It is reported that approximately 60% of the *Drosophila* genome is homologous to that of humans [26], and whether the other 40% genome is involved in this process remains unknown. Therefore, it would be significant if our findings could be verified in mammals or human cell lines in the future. Taken together, our results indicate that a high intake of fruits and vegetables rich in UA, such as apple, cranberry, daylily and so on, may protect against intestinal injury induced by environmental stresses.

## 5. Conclusions

In summary, UA could protect *Drosophila* adults from SDS-induced intestinal damage. The behavioral defects, morphological defects and the ROS production caused by SDS could be rescued by UA. Further studies indicated that UA could remarkably attenuate the SDS-induced excessive ISC proliferation and differentiation. Moreover, the protective effect of UA on UC is mediated by the JNK/JAK/STAT signaling pathway. Therefore, our data provided evidence for UA to be potentially developed as promising alternative functional foods and medicines for UC.

## Data Availability

The data presented in this study are available in the article and supplementary materials.

## Supplementary Materials

The following are available online at https://www.mdpi.com/article/10.3390/antiox11020426/s1, Figure S1: UA significantly restored SDS-induced nuclear enlargement..
