# Supplementary material for: Ursolic Acid Protects Sodium Dodecyl Sulfate-Induced Drosophila Ulcerative Colitis Model by Inhibiting the JNK Signaling
Source: Antioxidants (Basel). 2022 Feb 21;11(2):426. doi: 10.3390/antiox11020426 (PMC8869732; doi:10.3390/antiox11020426)
Supplement: Supplementary file 1 [file antioxidants-11-00426-s001.zip › antioxidants-1577818-supplementary.pdf]

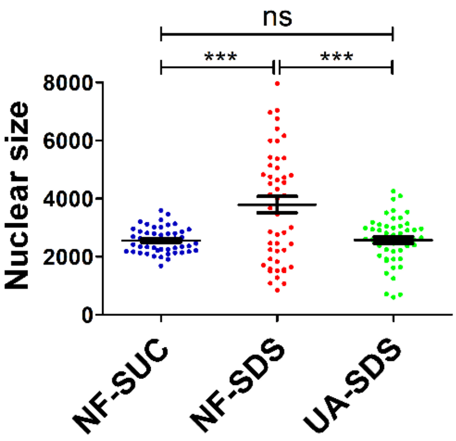

Figure S1. UA significantly restored SDS-induced nuclear enlargement. The UC model group (NF-SDS) enlarged nucleus size than control group (NF-SUC). 100 μM UA supplementation significantly rescued the SDS-induced aberrant nucleus morphology (n ≥ 50). ***$p < 0.01$, ns: no significant.
